# A Study on Rational Function Model Generation for TerraSAR-X Imagery

**DOI:** 10.3390/s130912030

**Published:** 2013-09-09

**Authors:** Akram Eftekhari, Mohammad Saadatseresht, Mahdi Motagh

**Affiliations:** 1 Department of Surveying and Geomatics Engineering, University of Tehran, Tehran 14395-515, Iran; E-Mail: msaadat@ut.ac.ir; 2 Helmholtz Centre Potsdam, GFZ German Research Centre for Geosciences, Potsdam 14473, Germany; E-Mail: Motagh@gfz-potsdam.de

**Keywords:** Rational Function Model, Range-Doppler model, SAR sensor orientation parameters, affine model

## Abstract

The Rational Function Model (RFM) has been widely used as an alternative to rigorous sensor models of high-resolution optical imagery in photogrammetry and remote sensing geometric processing. However, not much work has been done to evaluate the applicability of the RF model for Synthetic Aperture Radar (SAR) image processing. This paper investigates how to generate a Rational Polynomial Coefficient (RPC) for high-resolution TerraSAR-X imagery using an independent approach. The experimental results demonstrate that the RFM obtained using the independent approach fits the Range-Doppler physical sensor model with an accuracy of greater than 10^−3^ pixel. Because independent RPCs indicate absolute errors in geolocation, two methods can be used to improve the geometric accuracy of the RFM. In the first method, Ground Control Points (GCPs) are used to update SAR sensor orientation parameters, and the RPCs are calculated using the updated parameters. Our experiment demonstrates that by using three control points in the corners of the image, an accuracy of 0.69 pixels in range and 0.88 pixels in the azimuth direction is achieved. For the second method, we tested the use of an affine model for refining RPCs. In this case, by applying four GCPs in the corners of the image, the accuracy reached 0.75 pixels in range and 0.82 pixels in the azimuth direction.

## 1. Introduction

Sensor models are required to represent the functional relationship between 2D image space and 3D object space. In general, sensor models are classified into two categories: physical and generic models. The choice of a sensor model depends on a variety of factors, including the performance and accuracy required, the physical information of the acquisition system and the available control information [1].

In physical sensor models, the imaging process is described by parameters defining the position and orientation of a sensor with respect to an object-space coordinate system [2]. However, each imaging sensor has its own imaging system with various physical sensor models. This increases the difficulty of developing geometric processing software that is capable of handling multi-source remote sensing data. Thus, generic models are used in the processing of various remote sensing systems in a unified framework [3]. In a generic sensor model, a general function without any physical imaging process is used to represent the transformation between the image and the object space. There are four different generic sensor models defined in the Open Geospatial Consortium (OGC) papers[4]: the polynomial model, the Grid Interpolation Model (GIM), the Rational Function Model (RFM) and the Universal Image Geometry Model (UIGM). Previous studies have shown that accuracy is limited in polynomial and GIM models, which largely limits their application. The RFM uses ratios of polynomials to establish the relationship between the image coordinates and object coordinates, and it has been shown to be an ideal replacement for physical sensor models [5].

Accurate geolocation of Synthetic Aperture Radar (SAR) images depends on the quality and errors in SAR orbital data. Raggam *et al.* (2010) assessed the geolocation accuracy of Single-Look Slant-Range Complex (SSC) data from TerraSAR-X images by using a linear function with two parameters to describe the relationship between azimuth/range timing and column/line pixel values. The results showed that the Root Mean Square Error (RMSE) in the check points reached approximately 0.8 pixels in the column/line direction after applying a least-squares adjustment procedure [6]. In general, any uncertainty in physical parameters can affect the Rational Polynomial Coefficients (RPCs) that are calculated from them [7,8].

The focus of our study is different from the previous studies mentioned above in that we focus on SAR sensor parameter correction before RPC generation. In our procedure, four sensor parameters are first updated by using a different number, and distributed Ground Control Points (GCPs) are calculated, followed by RPCs. We test our methodology for producing RPCs for TerraSAR-X spotlight images by independent methods, and we calculate refined RPCs by using an independent approach. The sections of this article are described below. First, the basic concepts of physical and generic sensor models for SAR images are described. Then, in Section 2, the methodology of using an RF model for SAR images is explained, followed by an introduction to RPC adjustment models. Section 3 is devoted to the experiments and the results obtained by calculating RPCs in a terrain-dependent fashion and by applying two methods to calculate RPCs. A discussion is provided in Section 4.

## 2. Sensor Models

### 2.1. Physical Sensor Model for Spaceborne SAR

The Range–Doppler (RD) model is the most widely used physical sensor model for spaceborne SAR remote sensing systems [1]. This model is used for direct geolocation and transformation from the image space (azimuth line and range pixel) to the object space (latitude, longitude and height). Range and Doppler equations can also be used for indirect geolocation from the object space to the image space. In general, indirect geolocation is employed more frequently in practical applications.

### 2.2. Rational Function Model

The Rational Function Model (RFM) is defined as the ratio of two bi-cubic polynomials involving 39 parameters in any direction in a 3D form:

(1) $$R_{3D}\;(r,c)=\frac{\sum_{i=0}^{m}\sum_{j=0}^{n}\sum_{k=0}^{p}a_{\text{ijk}}X^{i}Y^{j}Z^{k}}{\sum_{i=0}^{m}\sum_{j=0}^{n}\sum_{k=0}^{p}b_{\text{ijk}}X^{i}Y^{j}Z^{k}}$$

In this equation, *a_ijk_* represents the polynomial coefficients called rational polynomial coefficients (RPC). The variables *r* and *c* are the normalised image coordinates [9].

Zhang *et al.* (2010) conducted a study about choosing the best number of coefficients for the RFM in SAR datasets, the experiments shown third-order polynomials with an unequal denominator got the best results [10]. Therefor we use third-order polynomials with an unequal denominator, as they are capable of modelling most of the distortion in the image space. Consequently, 39 terms are used, 20 terms for the numerator and 19 for the denominator. Thus, there are 78 unknown RPCs to be solved in the general RF model.

## 3. Methodology of RF Modelling

### 3.1. RPC Generation

**Figure 1.**
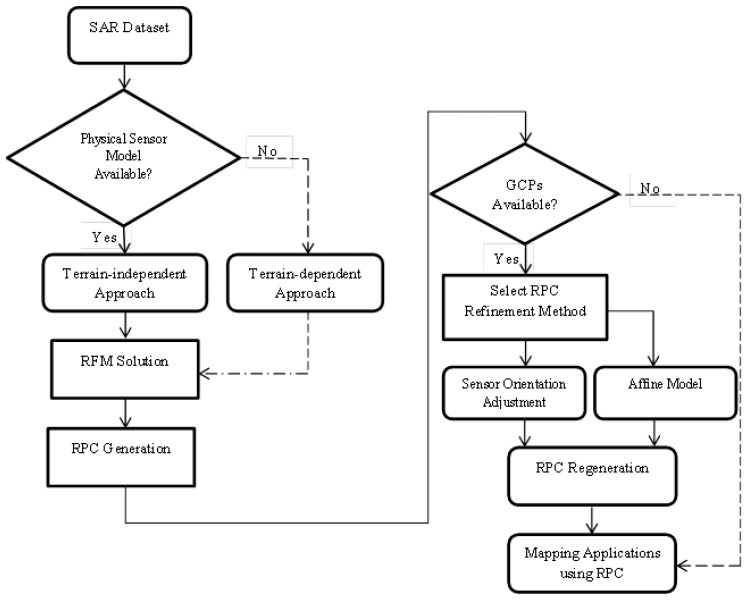
General workflow of developing a rational functional model [11]. SAR: synthetic aperture radar, RFM: rational function model, GCP: ground control point, RPC: rational polynomial coefficient.

The general workflow of developing the RF model used in this paper is shown in Figure 1. The procedure consists of three main processing steps. First, a grid of Control Points (CP) is established. These points can be either in real or virtual form, depending on the availability of SAR orbital information. In the second step, the RF model is solved by transforming it into a linear parametric model and determining the unknown RPCs. In this stage, the challenge is to select the appropriate regularisation method [11]. In the third processing step, RPCs are refined, the accuracy of the RPCs is checked and suitable models are fit to the data to improve precision.

### 3.2. RF Model Validation and Refinement

Because Range-Doppler equations and SAR sensor parameters are used for RPC generation, any error in the orbital data directly affects the coefficients. Therefore, it is important to analyse errors in the SAR sensor parameters.

The normal way to correct errors in the RPC is to use accurate Ground Control Points (GCPs) together with a suitable model, such as an affine transformation, to refine the RPCs [12]. Another method is to adjust the SAR parameters that affect RPC generation. In this method, sensor parameters are first adjusted, and then RPCs are calculated. In this study, we apply both methodologies to determine the appropriate method for correcting RPCs.

#### 3.2.1. RPC Model Refinement by Sensor Orientation Adjustment

In this method, physical sensor parameters that are involved in the generation RPCs are first updated, and then RPCs are calculated. The azimuth time for the first line, the Pulse Repetition Frequency (PRF), the range time for the first pixel and the Range Sampling Rate (RSR) in the azimuth and range directions are the four parameters that are updated in this approach [13]. To update these parameters, at least two GCPs are required. In this study, different combinations of control points are tested to determine the optimal number and proper distribution of GCPs for parameter adjustment.

#### 3.2.2. RPC Model Refinement with an Affine Model

Because the RF model is calculated from the physical imaging model without the aid of ground control points, errors in the direct measurement of sensor orientation can cause biases in the RPC mapping. The biases can be taken into account by using a bias-corrected RPC model, expressed as follows:

(2) $$\begin{array}{c} \text{line}+\Delta \text{l}=\frac{{\text{P}}_{1}(\text{X},\text{Y},\text{Z})}{{\text{P}}_{2}(\text{X},\text{Y},\text{Z})}\\ \text{sample}+\Delta \text{s}=\frac{{\text{P}}_{3}(\text{X},\text{Y},\text{Z})}{{\text{P}}_{4}(\text{X},\text{Y},\text{Z})} \end{array}$$

In these equations, **Δl** and **Δs** represent the differences between the measured and the nominal line, respectively, and the pixel coordinates. These deviations can generally be described as polynomials of the image line and sample coordinates [14]:

(3) $$\begin{array}{l} \Delta \text{l}={\text{A}}_{0}+{\text{A}}_{1\cdot }\text{line}+{\text{A}}_{2\cdot }\text{sample}+{\text{A}}_{3\cdot }{\text{line}}^{2}+{\text{A}}_{4\cdot }\text{line}.\text{sample}+{\text{A}}_{5\cdot }{\text{sample}}^{2}+\cdots \\ \Delta s={\text{B}}_{0}+{\text{B}}_{1\cdot }\text{line}+{\text{B}}_{2\cdot }\text{sample}+{\text{B}}_{3\cdot }{\text{line}}^{2}+{\text{B}}_{4\cdot }\text{line}.\text{sample}+{\text{B}}_{5\cdot }{\text{sample}}^{2}+\cdots \end{array}$$

here, **A_i_** and **B_i_** (**i** = 1, 2, 3 …) are the correction parameters in the bias-correction model. In this study, four sets of correction parameters are tested, and the results are compared:

(1) Case one. **A_0_** and **B_0_**, which model only the shift bias.
(2) Case two. **A_0_**, **A_1_**, **B_0_** and **B_1_**, which model the shift and time-dependent drift bias.
(3) Case three. **A_0_**, **A_1_**, **A_2_**, **B_0_**, **B_1_** and **B_2_**, which model the bias using an affine transformation model.

For more information about the physical meanings of the parameters, please see the relevant references [12,14].

## 4. Experiments and Results

### 4.1. Dataset

In this study, we use a TerraSAR-X Single-Look Slant-Range Complex (SSC) image as a test case for RF modelling. The image was acquired over the city of Jam, southern Iran, in spotlight mode. It was acquired on May 17 2011, in a descending orbit, covering an area of approximately 42 km^2^. The ground elevation in the study area is between 590 and 800 m. From the complex SSC product, an amplitude image was generated for use in this paper.

**Figure 2.**
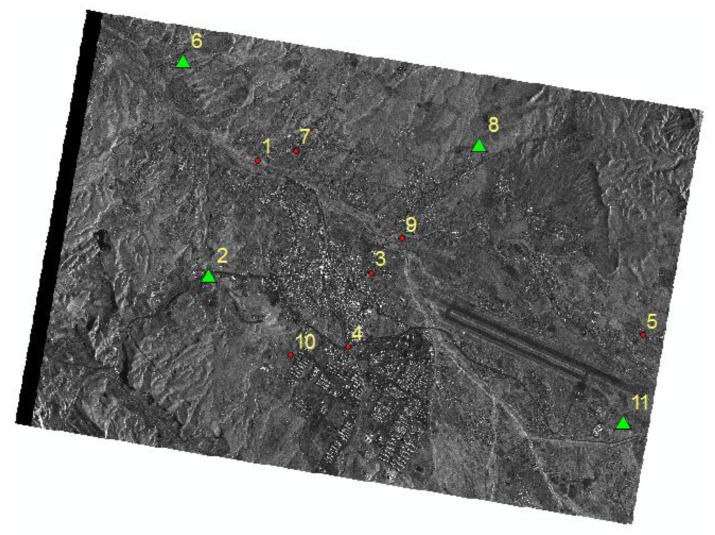
Distributions of ground control points (GCPs) and check points (CKPs) in the study area for a scenario involving five GCPs. Note: triangles represent the GCPs, and circles represent the CKPs.

**Figure 3.**
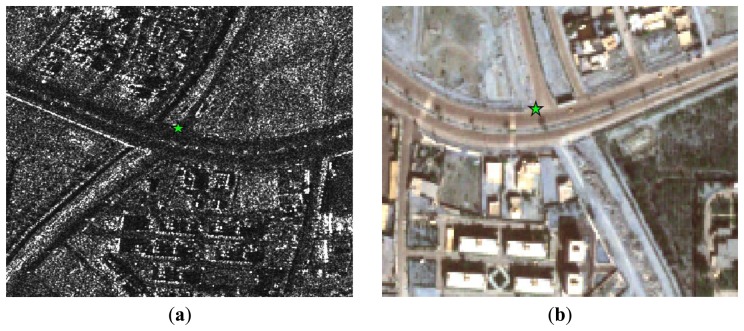
An example of a ground control point (GCP) selected in (**a**) the synthetic aperture radar (SAR) image and (**b**) the optical image.

In the experiment, a total of 11 distinct ground control points were measured by GPS. The accuracy of these points is less than 10 cm. The image coordinates of these 11 points were carefully measured up to a nominal accuracy of 1 pixel. All of these points were used as GCPs and check points (CKPs) for different configurations in the experiment. Figure 2 shows an example distribution of the GCPs and CKPs in the study area for one of the scenarios tested. Additionally, due to the absence of reflectors in the study area, natural targets such as road crossings, water bodies and the corners of buildings with low elevation are used as GCPs. Examples of these points are shown in Figure 3.

With these datasets, we conducted three experiments to provide a comprehensive evaluation of the RF model and to compare the performances of different bias correction methods. The experiments had the following aims:

1. To examine the RF model in an independent approach.
2. To examine the effects of GCP number and distribution on sensor orientation parameters and RPC generation with parameter adjustment.
3. To examine the use of an affine model for RPC refinement with different GCP numbers and distributions.

### 4.2. Results

#### 4.2.1. The RF Model in the Independent Approach

**Figure 4.**
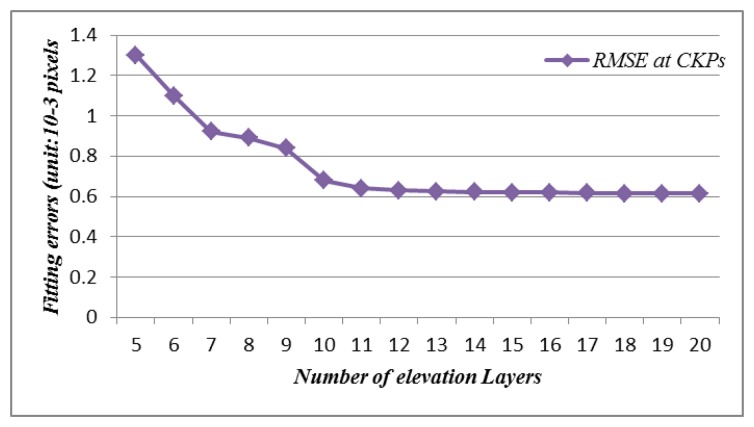
Plot of rational function modell (RFM) fitting errors versus number of elevation layers.

In the first experiment, the method described in Section 3 was applied to the SAR image to solve the RF model in an independent way. We took our lead from previous studies [8] and used a 20 × 20 horizontal grid size, with the number of elevation layers varying from 5 to 20 to allow us to choose the optimum elevation layer. Figure 4 shows the variation in planimetric RSME at CKPs *versus* the number of elevation layers. As the number of elevation layers increases, the variation of the fitting errors at control points (CNPs) becomes small (less than 10^−4^ pixel). For the CKPs, however, the fitting errors change little (less than 10^−4^ pixel) in the lines but change greatly in the pixels. In this study, we selected 10 layers as the optimum number of elevation layers and used the L-Curve regularisation method to determine the regularisation parameter. The results of the experiment are presented in Table 1.

#### 4.2.2. RPC Generation Using Updated SAR Sensor Orientation Parameters

**Figure 5.**
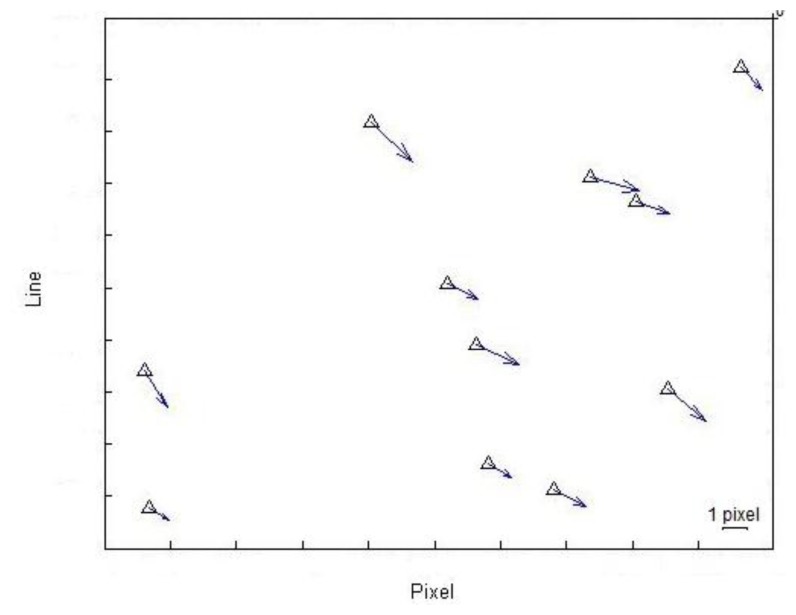
Discrepancies in the image space between the calculated rational polynomial coefficient (RPCs) and the true ground control points (GPCs).

In this section, we assess the accuracy of the original RPCs that were computed using the independent method by calculating the image coordinates for all GCPs using original RPCs. The results show a mean difference of 4.2 pixels in range and 3.7 pixels in azimuth between coordinates calculated by using original RPCs and true coordinates. These results demonstrate that there is a time-dependent drift error in the image orientation, as illustrated in Figure 5.

To account for the drift error, we adjust the physical sensor parameters to improve the accuracy of the RPCs. We use four physical parameters: azimuth time for the first line (ta_0_), Pulse Repetition Frequency (PRF), range time for first pixel (tr_0_) and Range Sampling Rate (RSR). These parameters are updated by ground control points. The initial values of these parameters are available in the SAR metadata. However, due to atmospheric effects and other disturbing factors, these parameters change with time. Therefore, using the initial values creates errors in imaging geometry and disturbs the conditions of the physical equations. The relationships between image coordinates and these four parameters are described by the following equations [13].

(4) $$t_{r}=t_{r0}+\frac{j-1}{2\text{RSR}}$$

(5) $$t_{a}=t_{a0}+\frac{i-1}{\text{PRF}}$$

In these equations, *t_r0_* and *t_r_* are the range time for the first pixel and the *j*th pixel, respectively, and RSR is the range sampling rate in Hz. *t_a0_* and *t_a_* are the azimuth time for first and *i*th line, and PRF is the pulse repetition frequency in Hz. Because there are four unknown parameters in the above equations, theoretically only two control points are needed to solve them. However, using more GCPs will increase the degrees of freedom in the system of equations and lead to a better estimation of the parameters. Of the 11 ground control points used in this study, several combinations of GCPs are extracted and used, and the remaining unknown parameters are calculated. For each scenario, there is a different number and distribution of control points, and the parameters are calculated, updated and used to calculate the RPCs. In Table 2, the results of our parameter estimation using different numbers of GCPs are given. After calculating the RPCs, the geographical coordinates and heights of the GCPs are converted to lines and pixels in image space with the direct RF model. Then, the computed values and actual values are compared, and the mean, standard deviation (STD) and Root Mean Square Error (RMSE) are calculated for each set. The results of these tests are presented in Table 3.

Note that the first columns in Tables 2 and 3 show the composition of the ground control points (GCPs), whose locations are shown in Figure 2. Columns 2 to 5 in Table 2 indicate the differences between the initial values and the calculated values of the four parameters, for different numbers and distributions of control points. The additional columns in Table 3 represent the errors (mean, STD, RMSE) in the control points (GCPs) and check points (CKPs) in the azimuth and range directions.

As is clear from the results in Table 3, when we use two control points, the best results occur when these two points are located in two opposite corners of the image (see the second row). When the two GCPs are located in the middle of image (third row) or in the range direction (first row), the results are not satisfactory. The results become more accurate when an additional control point is added, so by having two GCPs in the azimuth direction and one GCP in the range direction (all GCPs are in the corners of image) the error reaches 0.88 pixels in the azimuth and 0.69 pixels in the range direction (4th row of Table 3). By using four GCPs, the range error is increased, but the azimuth error is reduced so that, with the best distribution of control points (2, 6, 8, 11), the RMSE error is reduced by 3% compared to the case with only three control points (6, 8, 11). Finally, by using eight GCPs at the edge and corner of the image (the last column in Table 3), the azimuth error is reduced (18% less compared to the case with four GCPs), but the range error increases (68% greater compared to the case with four GCPs).

#### 4.2.3. RPC Refinement with the Affine Model

In this section, we use an affine model for RPC refinement. In this case, the coefficients of the affine model (A_0_–A_2_ and B_0_–B_2_ in Equation (2)) are calculated by using at least three GCPs, Δl and Δs are applied to all images points and the RPCs are regenerated. Lastly, the refined RPCs and the true coordinates are compared. Table 4 lists the results obtained with this methodology, using different scenarios for the GCPs. Like Table 3, the first column of Table 4 shows the different scenarios for the GCPs. The other columns represent the errors (mean, STD, RMSE) in the control points (GCPs) and check points (CKPs) in the azimuth and range directions.

As indicated in Table 4, using three GCPs to solve for the affine parameters yields an error of 1.93 pixels in range and 1.18 pixels in the azimuth direction (second row). By adding an additional control point such that there are four points in the corners of the image, the azimuth and range error are reduced to 0.84 and 0.75, respectively. However, when we select four GCPs at the edges of the image instead of the corners (fourth row in Table 4), the errors are slightly higher; the azimuth error increases by 12% and the range error increase by 27%. Increasing the number of control points in the centre of the image causes the azimuth error to be reduced (fifth row in Table 4) but increases the range error. We conclude that selecting four GCPs in the corners of image yields an optimal result.

## 5. Discussion

In this article, we applied the RF model to a spotlight TerraSAR-X image using an independent approach, with the aim of replacing the physical model with an appropriate generic sensor model. We showed that using the RF model with the independent method provides a good fit to the rigorous Range–Doppler model, with an accuracy of greater than 10^−3^ pixels in image space.

Two techniques were also tested for improving the accuracy of the RPCs. In the first method, SAR sensor orientation parameters are corrected by using a number of GCPs, and these are used to generate RPCs. In the second method, the RPCs are generated by using the initial values of the SAR sensor orientation parameters, and an affine model is used to refine the RPCs.

Experiments involving the first method indicate that, by using three GCPs in the image (GCPs 6, 8 and 11 in Table 3), the accuracy can reach 0.69 pixels in range and 0.88 pixels in the azimuth direction. Adding more GCPs will not affect the accuracy significantly. Thus, given the high cost of obtaining and measuring control points using GPS, selecting three GCPs is best in terms of accuracy and cost.

For the second method, in which an affine model is applied to refine the RPCs, selecting four GCPs in the corners of the image achieves the best accuracy. Using GCPs at the edges or centre of the image increases cost but does not improve accuracy.

Our implementations of the two methodologies for RPC adjustment show that increasing the number of GCPs from four to eight does not improve RPC adjustment accuracy, and it even causes a decrease in accuracy in the range direction. This is because GCPs have measurement errors of approximately 1 pixel in image space, and increasing the number of GCPs causes measurement errors to accumulate at the check points. Additionally, tests of different GCP scenarios show that it is not necessary to have GCPs in the centre of the image. By comparing the two methods, we find that they both produce similar results, with the first method requiring fewer control points. The main advantage of the first method, in which SAR sensor orientation parameters are corrected by using GCPs, is that the corrected parameters have interpretable physical meanings. In the second approach, in which affine coefficients are used to mitigate the accumulation of errors, the parameters are difficult to interpret from physical point of view.

Zhang *et al.* (2012) used an affine model for the RPC adjustment and by using five GCPs obtained a planimetric RMSE of 0.0668 pixels, which is approximately one order of magnitude better than what we obtained here. However, in that study, corner reflectors were placed during imaging, and the GCP measurement accuracy in the image space was one-sixteenth of a pixel [12]. In our experiment, we did not place any corner reflectors in the region. Instead, we used GCPs that were measured with 5 cm accuracy using GPS, and their positions were extracted from the SAR image with 1 pixel accuracy. This explains the order of magnitude difference in accuracy between this study and the one by Zhang *et al.* (2012). Our work demonstrates that, in areas where installing corner reflectors is difficult or impractical, achieving accuracy better than 1 pixel is still feasible by using GCPs to adjust RPCs from a SAR image, even if the GCPs are not very accurate.

## 6. Conclusions

This study demonstrates the use of a Rational Function Model for TerraSAR-X imagery. Although the Rational Function Model (RFM) has been widely used as an alternative to rigorous sensor models of high resolution optical imagery in photogrammetry and remote sensing geometric processing, not much has been done to assess its applicability for Synthetic Aperture Radar (SAR) image processing. We applied the RFM to a TerraSAR-X Single-Look Slant-Range Complex (SSC) image acquired over the city of Jam, southern Iran, which was acquired in spotlight mode with 11 control points that were measured using GPS. The accuracy of the control points was better than 10 cm. The RF model that we obtained with an independent approach fit the Range-Doppler physical sensor model with accuracy better than 10^−3^ pixels. However, errors in the physical SAR sensor parameters impair the absolute accuracy of the RPCs, and Ground Control Points (GCPs) should be used for RPC adjustment. Two methods were used for RPC adjustment. One uses updated physical SAR sensor orientation parameters, and the other uses an affine model for RPC refinement. The results show that, for both methods, using 3–4 GCPs is a good choice in terms of accuracy and cost for calculating RPCs with high accuracy. Additionally, the experiments indicate that control points at the corners of the image provide better accuracy than other placements. Planimetric accuracy reaches 1.12 pixels when we update the SAR sensor orientation parameters with three GCPs, and it reaches 1.11 pixels with the affine model method when four GCPs are used.

## Figures and Tables

**Table 1.** Results for rational function modelling by an independent method. RMSE: root mean square error, CNP: control point, CKP: check point.

**Regularisation Method**	**RMSE at CNPs (10^−3^Pixel)**			**RMSE at CKPs (10^−3^Pixel)**		
	
	**Line**	**Pixel**	**Planimetry**	**Line**	**Pixel**	**Planimetry**
L-Curve	0.031	0.035	0.047	0.042	0.049	0.065

**Table 2.** Changes in physical sensor parameters for different ground control points (GCP) scenarios. The locations of points listed in column 1 are shown in Figure 2. Δta_0_: azimuth time for the first line, PRF: pulse repetition frequency, Δtr_0_: range time for first pixel, RSR: range sampling rate.

**GCPs Num.**	**Δta_0_(ms)**	**ΔPRF (Hz)**	**Δtr_0_(ms)**	**ΔRSR (MHz)**
6,8	0.0309	8.602	2.27 × 10^−5^	218.43
6,11	0.2329	2.457	9.81 × 10^−5^	192.14
3,9	0.0811	5.582	3.88 ×10^−5^	273.11
6,8,11	0.3273	3.607	1.29 × 10^−5^	124.53
3,6,11	0.2497	2.252	1.20 × 10^−5^	114.87
2,6,8,11	0.3298	2.571	1.28 × 10^−5^	122.15
1,5,8,10	0.3138	1.711	1.22 × 10^−5^	117.68
1,5,8,9,10	0.2897	1.627	1.21 × 10^−5^	115.78
2,3,6,8,11	0.3286	2.614	1.356 × 10^−5^	130.12
1,2,3,5,6,8,10,11	0.2912	1.723	1.225 × 10^−5^	117.51

**Table 3.** The results of rational polynomial coefficient (RPC) adjustments by using synthetic aperture radar (SAR) sensor orientation correction in different ground control point (GCP) scenarios. The locations of points listed in column 1 are shown in Figure 2. CKP: check point, STD: standard deviation, RMSE: root mean square error.

**GCP** **Num.**	**Err. Pixel GCPs (pixels)**			**Err. Line GCPs (pixels)**			**Err. Pixel CKPs (pixels)**			**Err. Line CKPs (pixels)**		
			
	**Mean**	**STD**	**RMSE**	**Mean**	**STD**	**RMSE**	**Mean**	**STD**	**RMSE**	**Mean**	**STD**	**RMSE**
6,8	1.9 × 10^−4^	9.1 × 10^−5^	4.1 × 10^−4^	−4 × 10^−4^	1.2 × 10^−4^	4.1 × 10^−4^	0.54	2.13	2.19	−0.8	2.85	2.71
6,11	4.2 × 10^−5^	4.7 × 10^−5^	5.3 × 10^−5^	−4.2 × 10^−4^	2.3 × 10^−5^	4.3 × 10^−4^	−1.62	1.02	1.89	0.7	0.81	1.03
3,9	−3.8 × 10^−6^	1.7 × 10^−5^	1.2 × 10^−5^	−4.1 × 10^−4^	1.8 × 10^−5^	4.1 × 10^−4^	1.51	2.14	2.75	0.031	1.61	1.55
6,8,11	6.1 × 10^−5^	1.09	1.16	−3.9 × 10^−4^	0.74	0.61	−0.74	0.82	0.69	0.27	0.85	0.88
3,6,11	4.7 × 10^−6^	1.38	1.42	−4.4 × 10^−4^	0.75	0.76	−0.68	1.02	1.18	0.49	0.86	0.94
2,6,8,11	7.4 × 10^−5^	1.31	1.13	−4 × 10^−4^	0.88	0.76	−0.45	0.97	0.73	−0.35	0.65	0.80
1,5,8,10	1.8 × 10^−5^	0.81	0.69	−4.1 × 10^−4^	0.94	0.82	0.27	1.25	1.07	−0.57	0.61	0.82
1,5,8,9,10	−1.8 × 10^−4^	0.72	0.65	−4 × 10^−4^	0.9	0.81	0.16	1.37	1.26	−0.29	0.77	0.77
2,3,6,8,11	5 × 10^−5^	1.28	1.15	−4.1 × 10^−4^	0.77	0.68	0.12	1.05	0.97	−0.29	0.85	0.83
1,2,3,5,6,8,10,11	−1.8 × 10^−5^	1.01	0.94	−4.1 × 10^−4^	0.81	0.76	−0.39	1.48	1.23	−0.62	0.32	0.68

**Table 4.** The results of rational polynomial coefficient (RPC) refinement using the affine model in different ground control point (GCP) scenarios. The locations of the points in column 1 are shown in Figure 2. CKP: check point, STD: standard deviation, RMSE: root mean square error.

**Control** **Points**	**Err. Pixel GCPs (pixel)**			**Err. Line GCPs (pixel)**			**Err. Pixel CKPs (pixel)**			**Err. Line CKPs (pixel)**		
			
	**Mean**	**STD**	**RMSE**	**Mean**	**STD**	**RMSE**	**Mean**	**STD**	**RMSE**	**Mean**	**STD**	**RMSE**
6,8,11	−5.2 × 10^−4^	6.8 × 10^−4^	5.3 × 10^−4^	6.1 × 10^−4^	7.1 × 10^−4^	4.5 × 10^−4^	−0.87	2.00	2.17	0.86	1.17	1.38
3,6,11	−1.7 × 10^−4^	0.0017	0.0021	2.7 × 10^−4^	2.6 × 10^−4^	3.4 × 10^−4^	0.19	2.02	1.93	0.21	1.25	1.18
2,6,8,11	−2.5 × 10^−4^	1.30	1.02	4.7 × 10^−4^	0.75	0.31	−0.37	1.02	0.75	−0.48	0.74	0.84
1,5,8,10	−0.0012	0.56	0.48	−4.9 × 10^−4^	0.35	0.65	0.17	0.8	1.03	−0.15	0.99	0.96
1,5,8,9,10	−0.0011	0.52	0.47	−5.5 × 10^−4^	0.45	0.41	0.03	1.36	1.03	0.11	0.94	0.93
2 ,6,8,9,11	−1.5 × 10^−4^	1.28	1.14	4.8 × 10^−4^	0.66	0.59	0.11	1.05	0.96	−0.43	0.81	0.82
1,2,3,5,6,8, 10,11	−3.3 × 10^−4^	1.00	0.94	1.3 × 10^−4^	0.69	0.65	−0.19	1.48	1.22	−0.64	0.32	0.69
